# ZIF-67-Derived NiCo-Layered Double Hydroxide@Carbon Nanotube Architectures with Hollow Nanocage Structures as Enhanced Electrocatalysts for Ethanol Oxidation Reaction

**DOI:** 10.3390/molecules28031173

**Published:** 2023-01-25

**Authors:** Yixuan Li, Yanqi Xu, Cunjun Li, Wenfeng Zhu, Wei Chen, Yufei Zhao, Ruping Liu, Linjiang Wang

**Affiliations:** 1 College of Materials Science and Engineering, Guilin University of Technology, Guilin 541004, China; 2 Key Laboratory of New Technology for Processing Nonferrous Metals and Materials, Ministry of Education, Collaborative Innovation Center for Exploration of Nonferrous Metal Deposits and Efficient Utilization of Resources in Guangxi, Guilin University of Technology, Guilin 541004, China; 3 State Key Laboratory of Chemical Resource Engineering, Beijing University of Chemical Technology, Beijing 100029, China; 4 Beijing Institute of Graphic Communication, Beijing 102600, China

**Keywords:** layered double hydroxide, carbon nanotubes, sacrificial ZIF-67 template, hollow nanocage structure, electrocatalytic ethanol oxidation reaction

## Abstract

The rational design of efficient Earth-abundant electrocatalysts for the ethanol oxidation reaction (EOR) is the key to developing direct ethanol fuel cells (DEFCs). Among these, the smart structure is highly demanded for highly efficient and stable non-precious electrocatalysts based on transition metals (such as Ni, Co, and Fe). In this work, high-performance NiCo-layered double hydroxide@carbon nanotube (NiCo-LDH@CNT) architectures with hollow nanocage structures as electrocatalysts for EOR were prepared via sacrificial ZIF-67 templates on CNTs. Comprehensive structural characterizations revealed that the as-synthesized NiCo-LDH@CNTs architecture displayed 3D hollow nanocages of NiCo-LDH and abundant interfacial structure between NiCo-LDH and CNTs, which could not only completely expose active sites by increasing the surface area but also facilitate the electron transfer during the electrocatalytic process, thus, improving EOR activity. Benefiting from the 3D hollow nanocages and interfacial structure fabricated by the sacrificial ZIF-67-templated method, the NiCo-LDH@CNTs-2.5% architecture exhibited enhanced electrocatalytic activity for ethanol oxidation compared to single-component NiCo-LDH, where the peak current density was 11.5 mA·cm^−2^, and the j_f_/j_b_ value representing the resistance to catalyst poisoning was 1.72 in an alkaline environment. These results provide a new perspective on the fabrication of non-precious metal electrocatalysts for EOR in DEFCs.

## 1. Introduction

Direct ethanol fuel cells (DEFCs) have been regarded as a promising candidate for eco-friendly energy technology and have received considerable attention [1,2,3]. In the process of DEFCs, the ethanol oxidation reaction (EOR) is a sluggish step, taking place at the anode [4]. To date, platinum (Pt) and Pt-based nanoparticles have been developed as the most widely used anodic catalysts toward EOR in DEFCs because of their excellent activity [5,6,7,8,9,10]. However, the cost of Pt-based electrocatalysts is generally high, and sluggish kinetics, as well as relatively low durability, lead to limitations in their commercial application. In this respect, there is a wide commitment to developing non-noble metal electrocatalysts as Pt-based substitutes.

In recent years, two-dimensional (2D) materials, for example, layered double hydroxides (LDHs), spinel structures, transitional-metal sulfides, etc., have gained significant interest in the field of electrocatalysis and possess an incomparable structure superiority coming from the large surface area, rapid interfacial charge transfer rate, and fast electrocatalytic reaction rate [11,12,13,14,15]. LDHs are a kind of particularly promising 2D material and have received considerable attention for their electrocatalytic performance [16,17,18]. In particular, LDHs based on transition-metals (Fe, Co, Ni, and Mn) have attracted significant attention due to their superior EOR performance in alkaline media [19,20,21,22,23]. Nevertheless, plenty of the reported LDH-based electrocatalysts are still confronted with shortcomings, such as low surface area, poor conductivity, slow mass transport and electron transfer, etc. Furthermore, previous reports have revealed that the elaborately structural regulation of LDHs can increase the active surface area, in which the active sites can be in full contact with the electrolyte ions to guarantee fast mass transport and charge transfer, thus, improving the electrocatalytic performance [24].

As a kind of mature metal–organic skeleton, zeolite imidazolate frameworks (ZIFs) have been often used as providers of framework templates and metal feedstock for manufacturing energy storage and intermediate materials [25,26,27,28,29]. Especially, ZIF-67 composited by 2-methylimidazole and Co ions can supply trivalent ions and support frames to prepare LDH materials [30,31]. In addition, the electronic structure of LDHs can be adjusted by adding another metal element to improve electrocatalytic activity. Although using ZIF-67 templates as sacrifices can adjust the synthetic method and precisely control the morphology of LDHs, the poor conductivity of LDHs still has an impact on the electrocatalytic performance. This is usually solved by in situ growing LDHs on conductive matrices, for instance, carbon materials, metal foam, metal oxides, etc. [32,33,34]. Among these matrices, carbon nanotubes (CNTs) can achieve both high conductivity and a large specific area [32]. Therefore, the growth of LDHs on highly conductive CNTs with a large specific area is a key to achieving their enhanced electrocatalytic performance towards EOR.

Herein, a NiCo-layered double hydroxide@carbon nanotube (NiCo-LDH@CNT) architecture with a hollow nanocage structure as an electrocatalyst toward EOR was fabricated by sacrificing a ZIF-67 template on the conductive CNTs. The hollow nanocage structure of NiCo-LDH growing on CNTs could lead to completely exposed active sites by increasing the surface area, while the interfacial structure of NiCo-LDH@CNTs could facilitate the electron transfer during the electrocatalytic process, thus, improving the EOR activity. Hence, the electrocatalytic activity of NiCo-LDH@CNTs-2.5% toward EOR could be enhanced with the peak current density of 11.50 mA·cm^−2^ and the j_f_/j_b_ of 1.72 in an alkaline medium. This study will provide a new avenue toward the designing of efficient electrode materials for EOR.

## 2. Results and Discussion

**Scheme 1:**
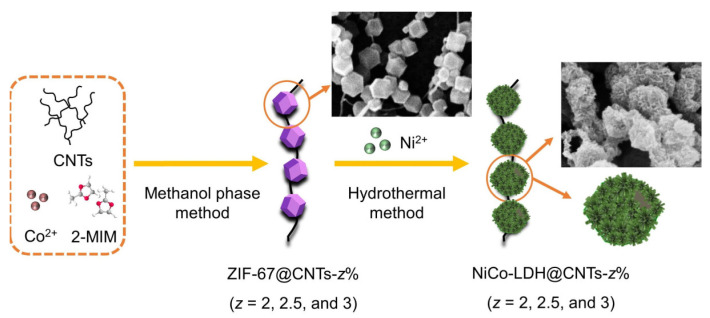
Schematic illustration for the synthetic process of NiCo-layered double hydroxide@carbon nanotubes (NiCo-LDH@CNTs-*z*%, *z* = 2, 2.5, and 3).

**Figure 1:**
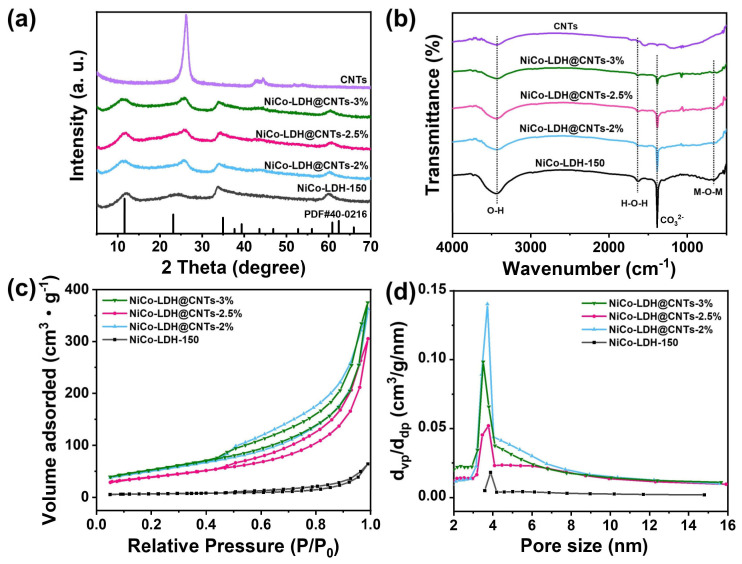
(**a**) XRD patterns and (**b**) FT-IR spectra of NiCo-LDH-150, NiCo-LDH@CNTs-*z*% (*z* = 2, 2.5, and 3), and CNTs, respectively. (**c**) N_2_-adsorption/desorption isotherms and (**d**) pore size distribution of the NiCo-LDH-150 and NiCo-LDH@CNTs-*z*% (*z* = 2, 2.5, and 3), respectively.

As shown in Scheme 1, the NiCo-layered double hydroxide@carbon nanotube (NiCo-LDH@CNT-*z*%, *z* = 2, 2.5, and 3) architectures with hollow nanocage structures were synthesized via sacrificial ZIF-67 templates on CNTs. To research the effect of feedstock molar ratios on the shape of the ZIF-67 template, three kinds of ZIF-67-*x* (*x* = 1/2, 1/3, and 1/4) nanocrystals were fabricated via a room-temperature method by altering Co(NO_3_)_2_·6H_2_O/2-methylimidazole molar ratios from 1/2 to 1/4 [35]. The XRD patterns and SEM images of ZIF-67-*x* (Figure S1) confirm that the uniform rhombic dodecahedron ZIF-67s (expressed as ZIF-67-1/4) can be prepared when the molar ratio is 1/4. Moreover, ZIF-67-template-derived NiCo-layered double hydroxides (NiCo-LDH-*y*, *y* = 50, 100, and 150) were fabricated by adjusting the additional amount of Ni(NO_3_)_2_·6H_2_O to pursue the optimal structure of NiCo-LDH [36]. When 150 mg Ni(NO_3_)_2_·6H_2_O was added, NiCo-LDH with a pure crystal structure and 3D flower-like structure (denoted as NiCo-LDH-150) could be fabricated, which was evidenced by the XRD patterns and SEM images (Figure S2). The structure of ZIF@CNT composites was related to the different amounts of CNTs; thus, ZIF-67@CNTs-*z*% (*z* = 2, 2.5, and 3) with different amounts of CNTs were fabricated [37]. The prepared processes of ZIF-67@CNTs-*z*% were similar to the synthesized processes of ZIF-67-*x*, except for the incorporation of pretreated CNTs [36]. The surface of CNTs was negatively charged by hydroxylation modification, and the resulting electrostatic repulsion caused them to disperse uniformly in solution. Therefore, with the addition of the Co(NO_3_)_2_·6H_2_O methanol solution, the deprotonation coordination between 2-methylimidazole and Co ions could quickly generate the anchoring of ZIF-67 clusters on CNTs under the condition of magnetic agitation. During subsequent growth, they gradually became ZIF-67 dodecahedrons, forming the ZIF-67@CNTs’ precursor. According to the XRD patterns and SEM images (Figure S3), the ZIF-67 with regular dodecahedrons were grown on CNTs, and the uniform bead string structure could be formed when the mass percentage of CNTs was 2.5%, while the ZIF-67 particles in the ZIF@CNTs-*z*% were unable to form hollow-nanocage-structured NiCo-LDHs when the mass percentages of CNTs were 2% and 3%. Subsequently, the NiCo-LDH@CNTs-*z*% (*z* = 2, 2.5, and 3) were prepared by the etching effect through solvent heat treatment. In the reaction, in situ etching and coprecipitation mechanisms dominated the formation of hollow structures and hydroxide nanosheets. Under solvothermal conditions, protons produced by Ni^2+^ hydrolysis can break the Co-2-methylimidazole bond and release Co^2+^ ions. At the same time, the released Co^2+^ ions diffused outward and coprecipitated with Ni^2+^ ions to form NiCo-LDH nanosheets on the surface of the ZIF-67 template. To corroborate the crystallography characteristics of the NiCo-LDH, NiCo-LDH@CNTs-*z*%, and CNTs (Figure 1a), an XRD measurement was performed. For NiCo-LDH-150, the crystal plane diffraction peaks at 11.4°, 22.8°, 34.1°, and 60.5° can be classified as (003), (006), (012), and (110) planes according to the LDH standard card (JCPDS 40-0216). The strong diffraction peak at 26.1° in the XRD pattern can be assigned to the (002) facet of CNTs [38]. As expected, all of the XRD patterns for NiCo-LDH@CNTs-*z*% samples match well with those reflections of NiCo-LDH-150 and CNTs, proving that NiCo-LDH was successfully grown on CNTs through the ZIF template.

Further, FT-IR spectroscopy was used to identify the species of the NiCo-LDH-150, NiCo-LDH@CNTs-*z*%, and CNTs. The broad absorption peak at 3430 cm^−1^ can be defined as the O-H tensile vibration of the H_2_O molecule, and the peak at 1630 cm^−1^ can be defined as the O-H bending vibration of the H_2_O molecule (Figure 1b). For the FT-IR spectra of NiCo-LDH-150 and NiCo-LDH@CNTs-*z*%, the absorption peaks at ~510, ~670, and ~1480 cm^−1^ are defined as the vibrations of metal-O-metal, metal-O, and CO_3_^2-^ bonds, respectively, suggesting that NiCo-LDH and CNTs are incorporated into NiCo-LDH@CNTs-*z*% [36]. The N_2_-adsorption/desorption isotherms and corresponding pore size distribution were carried out to research the specific surface area and the porosity of NiCo-LDH-150 and NiCo-LDH@CNTs-*z*% (Figure 1c,d). These materials all show typical type IV isotherms with H3-type hysteresis, suggesting a mesopore structure in the as-prepared samples [39]. The specific surface area of NiCo-LDH-150 (22.12 m^2^·g^−1^) is obviously lower than those of NiCo-LDH@CNTs-2% (182.35 m^2^·g^−1^), NiCo-LDH@CNTs-2.5% (140.89 m^2^·g^−1^), and NiCo-LDH@CNTs-3% (191.21 m^2^·g^−1^) (Table S1). This result robustly demonstrates that the addition of CNTs effectively reduces the aggregation of NiCo-LDH nanosheets and, hence, provides a larger specific surface area for NiCo-LDH@CNTs-*z*%, which is beneficial to fully expose active sites to participate in the EOR. According to the BJH calculation, the distinct peaks related to the pore width of 3.88, 3.72, 3.77, and 3.50 nm for NiCo-LDH-150, NiCo-LDH@CNTs-2%, NiCo-LDH@CNTs-2.5%, and NiCo-LDH@CNTs-3%, respectively, can be detected. The suitable pore size distribution of the samples will ensure the facile mass transport and charge transfer during the EOR process. It is commonly recognized that a large number of mesopores can form a more open structure, which is of benefit to the mass transfer and, thus, improves the EOR performance of the materials.

**Figure 2:**
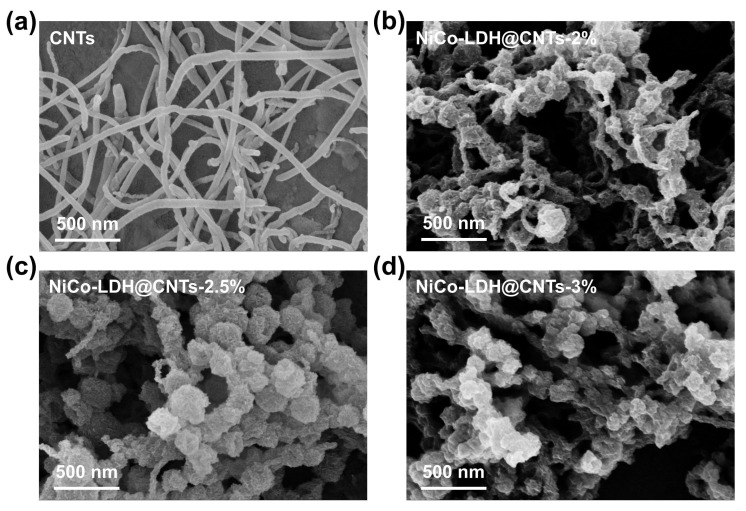
SEM images of (**a**) CNTs, (**b**) NiCo-LDH@CNTs-2%, (**c**) NiCo-LDH@CNTs-2.5%, and (**d**) NiCo-LDH@CNTs-3%.

Figure 2 shows the detailed morphological characterizations of CNTs and NiCo-LDH@CNTs-*z*%. The SEM image shows that CNTs display a smooth 1D tubular structure in Figure 2a, while the SEM images recorded for NiCo-LDH@CNTs-*z*% reveal that NiCo-LDH nanosheets derived from ZIF-67 template wrapped around the CNTs (Figure 2b–d). Notably, the NiCo-LDH nanosheets in the NiCo-LDH@CNTs-2.5% architecture exhibit a 3D hollow nanocage structure, leading to gains in larger surface areas than the spherical bulk morphology of NiCo-LDH-150 (Figure S2d). Such an open nanostructure of NiCo-LDH@CNTs-2.5% will expose electrochemical active sites and make the electrolyte accessible, thereby improving the performance of EOR. In addition, the real percentage of Ni and Co in the NiCo-LDH-150 and NiCo-LDH@CNTs-*z*% were measured by the EDS test, and the results are shown in Figure S4. The atomic ratios of Ni/Co for NiCo-LDH-150, NiCo-LDH@CNTs-2%, NiCo-LDH@CNTs-2.5%, and NiCo-LDH@CNTs-3% could be calculated to be 2.32, 2.52, 2.77, and 2.12, respectively.

**Figure 3:**
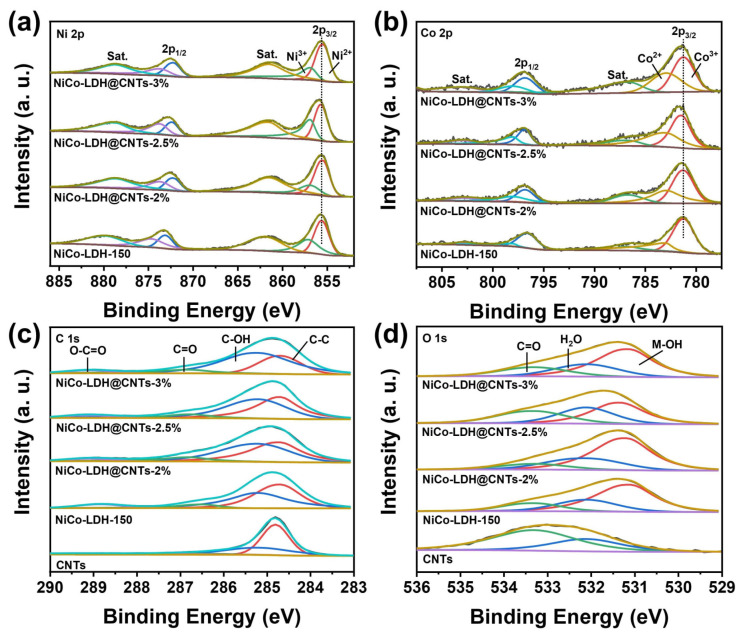
(**a**) Ni 2p and (**b**) Co 2p XPS spectra of NiCo-LDH-150 and NiCo-LDH@CNTs-*z*% (*z* = 2, 2.5, and 3). (**c**) C 1s and (**d**) O 1s XPS spectra of CNTs, NiCo-LDH-150, and NiCo-LDH@CNTs-*z*% (*z* = 2, 2.5, and 3).

XPS was used to study the electronic mutual effect and charge transfer between the CNTs and NiCo-LDH of the NiCo-LDH@CNTs-*z*% architecture. The XPS spectra in Figure S5 demonstrate the presence of Ni, Co, C, and O in the samples of NiCo-LDH@CNTs-*z*% and NiCo-LDH-150. The high-resolution Ni 2p spectra for NiCo-LDH@CNTs-*z*% and NiCo-LDH-150 (Figure 3a) exhibit two peaks of Ni 2p_3/2_ and Ni 2p_1/2_ combined with two satellites at 861.8 and 879.9 eV. For NiCo-LDH-150, two peaks classified as Ni^2+^ are fitted at 855.8 and 874.1 eV, and two peaks classified as Ni^3+^ are fitted at 857.4 and 875.8 eV [40,41]. The Ni^3+^/Ni^2+^ ratio in the NiCo-LDH-150 was 0.39 (Table S2), which was lower than those in NiCo-LDH@CNTs-2% (0.65), NiCo-LDH@CNTs-2.5% (0.82), and NiCo-LDH@CNTs-3% (0.43). This result indicates that Ni was oxidized after growing on CNTs and showed the highest valence state in NiCo-LDH@CNTs-2.5%. Two distinct peaks of Co 2p_3/2_ and Co 2p_1/2_ appearing at 781.4 and 796.8 eV accompanied by two broad satellite peaks can be seen in the Co 2p spectra (Figure 3b). For NiCo-LDH-150, two fitting peaks at 781.3 and 796.8 eV are classified as Co^3+^, and two fitting peaks at 783.1 and 798.2 eV are classified as Co^2+^ [31,32]. From the spectra of NiCo-LDH@CNTs-*z*%, the binding energy of Co 2p_3/2_ and Co 2p_1/2_ transferred to higher binding energy, and the Co^3+^/Co^2+^ ratio in the NiCo-LDH@CNTs-2.5% was the lowest (Table S2). The shifting binding energy of Ni 2p and Co 2p for NiCo-LDH@CNTs-*z*% proves a strong electronic mutual effect between CNTs and NiCo-LDH. The fitting peaks at 284.8, 285.3, and 286.8 eV in the C 1s spectra of CNTs can be assigned to C-C, C-OH, and C=O bonds (Figure 3c) [42]. In addition to the fitting peaks for C-C, C-OH, and C=O bonds, an additional peak at 289.4 eV ascribed to O-C=O was discernible in the C 1s spectra of NiCo-LDH-150. As for the C 1s spectra of NiCo-LDH@CNTs-*z*%, all fitting peaks attributed to CNTs and NiCo-LDH were observable, indicating that CNTs and NiCo-LDH are the toughest composed. As depicted in Figure 3d, the O 1s spectra of CNTs can be fitted as two peaks at 532.2 eV and 533.4 eV and assigned to C=O and H_2_O respectively, while the O 1s spectra of NiCo-LDH@CNTs-*z*% and NiCo-LDH-150 can be deconvoluted into three peaks (531.2, 532.2, and 533.4 eV) dovetailing with C=O, H_2_O, and M-OH bonds, respectively.

**Figure 4:**
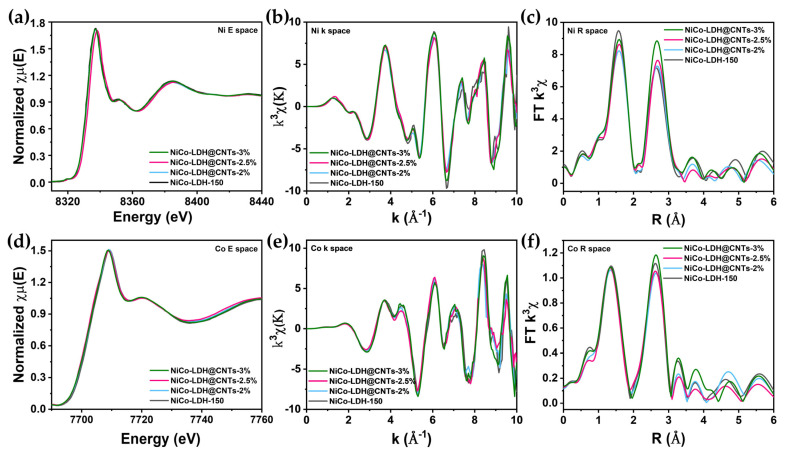
(**a**) Ni K-edge XANES spectra, (**b**) Ni K-edge EXAFS oscillation functions k^3^χ(k), (**c**) the magnitude of k^3^-weighted FT of the Ni K-edge extended EXAFS spectra, (**d**) Co K-edge XANES spectra, (**e**) Co K-edge EXAFS oscillation functions k^3^χ(k), and (**f**) the k^3^-weighted FT magnitude of the Co K-edge extended EXAFS spectra of NiCo-LDH-150 and NiCo-LDH@CNTs-*z*% (*z* = 2, 2.5, and 3).

The local electronic structures and coordination environments of the as-synthesized catalysts were clearly confirmed by X-ray absorption fine structure (XAFS) spectra. As seen in Figure 4a, the Ni K-edge X-ray absorption near-edge structure (XANES) spectra for NiCo-LDH@CNTs-*z*% and NiCo-LDH-150 show comparable spectra. Noteworthily, the Ni K-edge absorption position of NiCo-LDH@CNTs-2.5% shifts to a high-energy direction, which proves that the valence state of Ni in NiCo-LDH@CNTs-2.5% is higher than that of the other samples and reconciles the XPS results. From the Ni K-edge k-space plot (Figure 4b), the oscillation spectra of NiCo-LDH@CNTs-*z*% are similar to NiCo-LDH-150, indicating that the LDH structure still maintains its structure. The Ni Fourier-transformed extended X-ray absorption fine structure (FT-EXAFS) spectra of NiCo-LDH@CNTs-*z*% and NiCo-LDH-150 (Figure 4c) exhibit two main peaks assigned to the Ni-O shell at ~1.6 Å and the Ni-M (M = Co or Ni) shell at ~2.7 Å, respectively [30,32]. The peak intensity of the Ni-O shell in NiCo-LDH-150 is higher than those of NiCo-LDH@CNTs-*z*%. As the content of CNTs increases from 2% to 3%, the peak strength of NiCo-LDH@CNTs-*z*% decreases first and then increases, which may be ascribed to the distortion of the LDH structure after in situ growing on CNTs. Interestingly, the peak strength of the Ni-M shell also changes with the change in carbon nanotube content. When the content increases, the peak strength increases, showing an increase in the Ni-M coordination number. In the Co K-edge XANES spectra (Figure 4d), a slight shift in the absorption edge for NiCo-LDH@CNTs-2.5% toward the low-energy direction can be observed, proving that the valence state of Co in NiCo-LDH@CNTs-2.5% is the lowest among all as-prepared samples. Compared with NiCo-LDH-150, the Co K-edge k^3^χ(k) spectra of NiCo-LDH@CNTs-*z*% show a similar amplitude at high k values, and the subtle difference in the Co atom coordination environment can be further proved (Figure 4d). The amplitude of the Co K-edge k^3^χ(k) oscillation in NiCo-LDH@CNTs-*z*% is comparable to NiCo-LDH-150 (Figure 4e). The Co K-edge R-space spectra of NiCo-LDH@CNTs-*z*% and NiCo-LDH-150 also show two main peaks assigned to the Co-O shell at ~1.60 Å and the Co-M (M = Ni or Co) shell at ~2.70 Å (Figure 4f) [30,43]. Interestingly, the intensity of the Co-O coordination peaks is basically unchanged, while the peak intensity of the second shell Co-M varied with the number of CNTs added. With the increase in CNT content, the peak intensity also increases gradually, indicating that the coordination number of Co-M increases. Overall, the XAFS results provide strong evidence for the valence state and coordination structure of NiCo-LDH@CNTs-*z*% that will affect the electrocatalytic performance of EOR, as discussed below.

**Figure 5:**
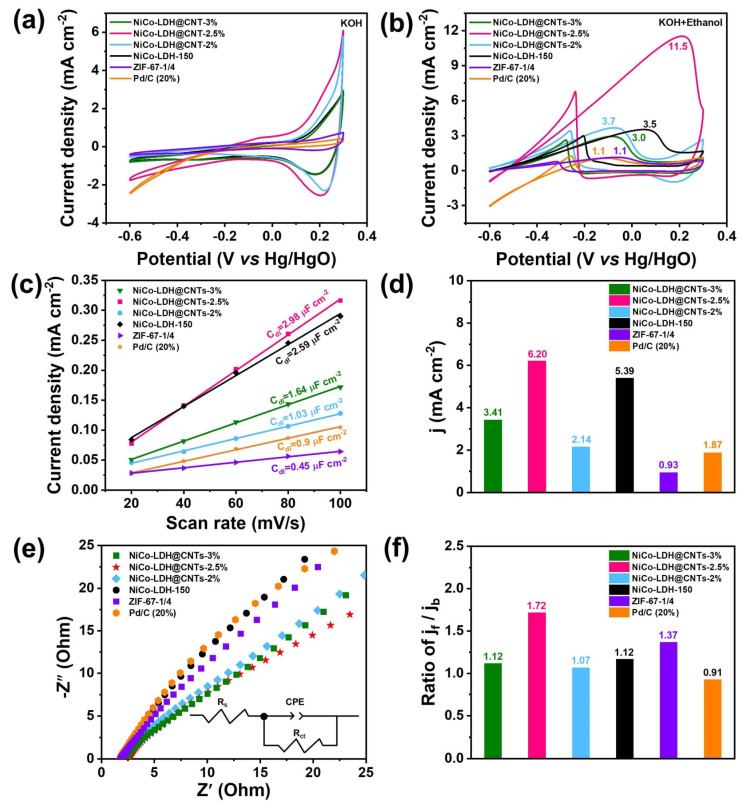
The CV curves of Pd/C (20%), ZIF-67-1/4, NiCo-LDH-150, and NiCo-LDH@CNTs-*z*% (*z* = 2, 2.5, and 3) in (**a**) 1.0 M KOH and (**b**) 1.0 M KOH and 1.0 M ethanol solution saturated with nitrogen at 20, 40, 60, 80, and 100 mV s^−1^ sweep rate. (**c**) C_dl_, (**d**) ECSA, (**e**) EIS spectra, and (**f**) j_f_/j_b_ of Pd/C (20%), ZIF-67-1/4, NiCo-LDH-150, and NiCo-LDH@CNTs-*z*% (*z* = 2, 2.5, and 3), respectively.

In the mixed electrolyte of 1.0 M KOH plus 1.0 M ethanol saturated with N_2_, the electrocatalytic performance of the as-prepared NiCo-LDH@CNTs-*z*% for EOR was studied using a standard three-electrode system. The EOR performance was evaluated based on the cyclic voltammetry (CV) curves recorded at the range of −0.6 to 0.3 V, as shown in Figure 5a,b. It can be found that NiCo-LDH@CNTs-2.5% exhibits the highest intensities and largest active areas among all the electrocatalysts in the 1.0 M KOH electrolyte saturated with N_2_, suggesting NiCo-LDH@CNTs-2.5% has more active sites and higher utilization efficiency (Figure 5a). The EOR activities of the electrocatalysts were measured in the KOH (1.0 M) solution saturated with N_2_ containing ethanol (1.0 M). The forward peak current density was an important parameter for kinetic quantitative analysis of electrocatalysts’ activity, and the higher the peak current density indicates the stronger the EOR activity of the electrocatalysts [44,45]. The peak current density of NiCo-LDH@CNTs-3%, NiCo-LDH@CNTs-2.5%, NiCo-LDH@CNTs-2%, NiCo-LDH-150, ZIF-67-1/4, and Pd/C (20%) can be calculated to be 3.0, 11.5, 3.7, 3.5, 1.1, and 1.1 mA·cm^−2^, respectively (Figure 5b). Furthermore, the EOR peak potential in the negative-to-positive sweep direction was also regarded as another parameter to assess the EOR activity, where the higher the potential, the better the performance [46]. As shown in the Figure 5b, the corresponding peak potential of NiCo-LDH@CNTs-2.5% was 0.21 V, which was much larger than those of NiCo-LDH@CNTs-3%, NiCo-LDH@CNTs-2%, NiCo-LDH-150, ZIF-67-1/4, and Pd/C (20%). As such, the NiCo-LDH@CNTs-2.5% shows the highest electrochemical EOR activity among these electrocatalysts. So as to study the surface effect of the NiCo-LDH@CNTs-*z*% on the EOR performance, CV curves were used to measure the double-layer capacitance (C_dl_) in a non-faradaic region of −0.24 to −0.14 V at different scan rates (Figure 5c and Figure S6). The values of C_dl_ for NiCo-LDH@CNTs-3%, NiCo-LDH@CNTs-2.5%, NiCo-LDH@CNTs-2%, NiCo-LDH-150, ZIF-67-1/4, and Pd/C (20%) in the 1.0 M KOH plus 1.0 M ethanol mixed solution saturated with N_2_ can be calculated to be 1.64, 2.98, 1.03, 2.59, 0.45, and 0.9 μF·cm^−2^, respectively, indicating NiCo-LDH@CNTs-2.5% has abundant active sites. Moreover, the electrochemically active surface areas (ECSAs) of NiCo-LDH@CNTs-3%, NiCo-LDH@CNTs-2.5%, NiCo-LDH@CNTs-2%, NiCo-LDH-150, ZIF-67-1/4, and Pd/C (20%) can be evaluated to be 3.41, 6.20, 2.14, 5.39, 0.93, and 1.87 mA·cm^−2^, respectively (Figure 5d). Furthermore, the ECSA value of NiCo-LDH@CNTs-2.5% is the largest, suggesting the contact area between the active site on the electrocatalyst and the electrolyte is the largest. The electrochemical impedance of NiCo-LDH@CNTs-*z*% is preferable for the transportation of electrons than that of NiCo-LDH-150, as demonstrated by electrochemical impedance spectroscopy (EIS) measured at open-circuit potential conditions (Figure 5e). Noteworthily, NiCo-LDH@CNTs-2.5% showed the smallest semicircle among the electrocatalysts, suggesting the facilitated electron transfer rates, thus, improving EOR activity. In addition, the equivalent circuit diagram in Pd/C (20%), ZIF-67-1/4, NiCo-LDH-150, and NiCo-LDH@CNTs-*z*% is shown in Figure 5e, which reveals the resistance of electrolyte (Rs) and charge-transfer resistance (Rct) of electrocatalysts for EOR [45,47]. Compared to Pd/C (20%), ZIF-67-1/4, NiCo-LDH-150, NiCo-LDH@CNTs-2%, and NiCo-LDH@CNTs-3%, NiCo-LDH@CNTs-2.5% showed the lowest Rct value of 28.51 Ω (Table S3), confirming that the superior catalytic activity of NiCo-LDH@CNTs-2.5% was mainly due to fast electron transfer. These can be attributed to the increase in CNT contents, which leads to the better conductivity of materials. Moreover, the ratio of j_f_/j_b_ represents the resistance of the electrocatalysts to poisoning, where j_f_ and j_b_ are the specific activity peak currents during forward and reverse scanning, respectively [48]. The NiCo-LDH@CNTs-2.5% has the highest j_f_/j_b_ ratio of 1.72 among the samples, indicating the best anti-poisoning ability (Figure 5f). This result may be related to the fact that the synergistic effect between CNTs and NiCo-LDH and the 3D hollow cage structure formed in the NiCo-LDH@CNTs-2.5% is more conducive to ion transport than other samples.

To research the long-term stability of the NiCo-LDH@CNTs-2.5% in the EOR process, the CA curves were recorded at -0.3 V (*vs*. Hg/HgO) in the N_2_-saturated 1.0 M KOH plus 1.0 M ethanol solution for 8000 s (Figure S7). After 8000 s, NiCo-LDH@CNTs-2.5% still had a high current density, which was higher than the current density of NiCo LDH-150. XRD, XPS, and XAFS techniques were applied to determine the structural stability of the NiCo-LDH@CNTs-2.5% after the reaction (denoted as Re-NiCo-LDH@CNTs-2.5%, Figure S8). There were no significant changes by comparing the XRD patterns of Re-NiCo-LDH@CNTs-2.5% and NiCo-LDH@CNTs-2.5%, suggesting the crystal structure stability of the material was confirmed. XPS and XAFS spectra of Re-NiCo-LDH@CNTs-2.5% show that the valence state of Ni species decreases while the valence state of Co species increases, suggesting the Ni and Co species participate in the EOR process (Figure S8b–i and Table S2). Especially, a small change in the Ni atom coordination environment could be detected, while obvious changes had taken place in the Co atom coordination environment, indicating Co species play an authentic electrocatalytic role. The above series of research results show that NiCo-LDH@CNTs-2.5% has better EOR activity, anti-poisoning ability, and durability compared to NiCo-LDH-150. This is mainly due to the synergy between CNTs and NiCo-LDH, as well as the structural advantages of the 3D hollow nanocage structure.

The EOR reaction pathway over NiCo-LDH@CNTs-2.5% was simply proposed based on the previous reports and is shown in Figure S9 [10,49,50]. According to the above experimental data, the EOR may take place on the NiCo-LDH surface, and Co species play an authentic electrocatalytic role. At stage 1, ethanol was adsorbed on the NiCo-LDH surface of NiCo-LDH@CNTs-2.5%, and the dehydrogenation happened to generate *CH_3_CH_2_O and *CH_3_CHOH. Thereafter, in stage 2, the *CH_3_CO was formed by the reaction of *CH_3_CH_2_O and *CH_3_CHOH with the *OH species that were dissociated by the adsorbed water on the electrode surface. *CH_3_CO was determined as the key intermediate before the divergence to either the C1 to CO_2_ via C−C bond-breaking or the C2 route to CH_3_COOH via a hydroxylation process. If the *CH_3_CO follows the C1 route, the C−C bond will be broken into *CO and *CH_3_. The *CO group will go through a complete oxidation process to CO_2_ via dehydrogenation and hydroxylation processes, while the *CH_3_ will have a more complex oxidation process to CO first and then to the final CO_2_. Nevertheless, if the *CH_3_CO follows the C2 route, the as-formed *CH_3_COOH by hydroxylation can desorb from the surface to form CH_3_COOH. Therefore, the different products of EOR over the electrocatalyst may co-exist.

## 3. Materials and Methods

### 3.1. Chemicals and Reagents

Ni(NO_3_)_2_·6H_2_O, NaOH, KOH, H_2_SO_4_, HNO_3_, CH_3_CH_2_OH, and urea were purchased from Guangdong Xilong Chemical Reagent Co., Ltd. (Shantou, China). Carbon nanotubes (CNTs), Co(NO_3_)_2_·6H_2_O, and 2-methylimidazole were bought from Shanghai Aladdin Bio-Chem Technology Co., Ltd. (Shanghai, China). Nafion solution (5%) was purchased from Shanghai For-mat New Energy Technology Co., Ltd. (Shanghai, China). Nickel foam (NF) was bought from Changde Liyuan New Material Co., Ltd. (Changde, China).

### 3.2. Instrumentations

X-ray diffraction (XRD) patterns were characterized by X’ Pert PRO with a Cu Kα radiation source (λ=1.5405˚A). Fourier-transform infrared spectroscopy (FT-IR) spectra were recorded with Thermo Nicolet NEXUS 670 in the range of 400-4000 cm^−1^. The Brunauer–Emmett–Teller (BET) surface area was measured on a TriStar Ⅱ 3200. Field-emission scanning electron microscopy (SEM) was performed by Hitachi S4800 SEM. X-ray photoelectron spectroscopy (XPS) measurements were performed on Thermo Scientific K-Alpha X-ray photoelectron spectrometer. The X-ray absorption structure (XAS) was collected at the 1W1B beamlines of the Beijing Synchrotron Radiation Facility (BSRF), China.

### 3.3. Synthesis of NiCo-LDH@CNTs-z% (z = 2, 2.5, and 3)

The ZIF-67-*x* (*x* = Co^2+^/2-methylimidazole molar ratios, *x* = 1/2, 1/3, and 1/4) nanocrystals were fabricated via a room-temperature method [35]. Typically, 2 mmol Co(NO_3_)_2_·6H_2_O was dispersed in 40 mL methanol to obtain a clear solution, and then, different molar amounts of 2-methylimidazole (4, 6, and 8 mmol) were added into the dispersed solution. The mixed solutions were stirred at room temperature (RT) for 2 h, and the sediment of ZIF-67-*x* (*x* = 1/2, 1/3, and 1/4) was centrifuged, washed with methanol, and dried at 60 °C for 10 h.

The ZIF-67-template-derived NiCo-layered double hydroxide (NiCo-LDH-*y*, *y* = quality of Ni(NO_3_)_2_·6H_2_O, *y* = 50, 100, and 150) was fabricated through the reported method [28]. In the typical process, added ZIF-67-1/4 (30 mg) was dispersed evenly in 30 mL ethanol, following which 50, 100, and 150 mg Ni(NO_3_)_2_·6H_2_O were added in. The mixture was stirred at RT for 30 min, transferred to Teflon-lined stainless-steel autoclaves and heated to 80 °C, and then reacted for 2 h. The NiCo-LDH-*y* (*y* = 50, 100, and 150) were precipitated, centrifuged, washed with ethanol, and dried at 60 °C for 10 h.

The ZIF-67-template-derived NiCo-LDH@CNTs-*z*% (*z* = CNTs/(Co(NO_3_)_2_·6H_2_O + 2-methylimidazole) wt%, *z* = 2, 2.5, and 3) were prepared by the following steps [36]: The CNTs were pretreated by sonication using a mixed acid solution of H_2_SO_4_ and HNO_3_ (3/1 (v/v)). After ultrasonic treatment at RT for 1.5 h, the above mixture was transferred into Teflon-lined stainless-steel autoclave, and then, the autoclave was heated to 85 °C and reacted for 5 h. Then, the treated CNTs were washed many times with deionized water until pH ~ 7. The 24.7, 30, and 37.1 mg pretreated CNTs were dispersed in 40 mL methanol containing Co(NO_3_)_2_·6H_2_O (2 mmol) and 2-methylimidazole (8 mmol), and then, the mixtures were stirred at RT for 2 h to ensure they dispersed evenly. The obtained ZIF-67@CNTs-*z*% (*z* = 2, 2.5, and 3) were centrifuged, washed with methanol, and dried at 60 °C for 10 h. Subsequently, the ZIF-67@CNTs-*z*% were dispersed in 30 mL ethanol containing 150 mg Ni(NO_3_)_2_·6H_2_O, and the mixtures were transferred into Teflon-lined stainless-steel autoclaves and heated to 80 °C and then reacted for 2 h. The obtained precipitates of NiCo-LDH@CNTs-*z*% (*z* = 2, 2.5, and 3) were centrifuged, washed with ethanol, and dried at 60 °C for 10 h.

### 3.4. Electrochemical Measurements

Electrochemical measurements were performed on an electrochemical workstation (CHI 660E, CH Instruments Inc., Shanghai) using a three-electrode mode. NF (5 mm*5 mm) coated with the prepared sample was used as the working electrode, and a Hg/HgO electrode was used as the reference electrode, while the Pt sheet was used as the counter electrode. The working electrode was fabricated as follows: 5 mg of catalyst was dispersed in 1 mL of ethanol solution and sonicated for 15 min, then 50 μL of the Nafion was dripped and sonicated for 15 min to form a uniformly dispersed slurry, which was subsequently coated on NF surface and dried for 6 h. EOR was measured in 1.0 M KOH plus 1.0 M ethanol solution saturated with nitrogen by cyclic voltammetry (CV) in the potential range from -0.6 to 0.3 V with 50 mV s^−1^ sweep rate at room temperature. The electrochemical impedance spectra (EIS) were measured at open circuit potential from 100 kHz to 0.01 Hz. The chronoamperometric (CA) curves were measured in 1.0 M KOH plus 1.0 M ethanol solution saturated with nitrogen at a potential of 0.3 V at room temperature.

## 4. Conclusions

In summary, NiCo-LDH@CNTs-2.5% with hollow nanocage structures as electrocatalysts toward EOR were successfully fabricated via a sacrificial ZIF-67 template on conductive CNTs. According to the results of XRD, BET, SEM, XPS, and XAFS, the in situ growing of NiCo-LDH with hollow nanocage structures on the conductive CNTs through sacrificing ZIF-67 template made NiCo-LDH nanosheets disperse uniformly, avoided agglomeration, and ensured the full exposure of surface-active sites. Moreover, a stable interface was fabricated between NiCo-LDH and CNTs and accelerates the interface electron transfer. As a result, NiCo-LDH@CNTs-2.5% showed enhanced EOR activity with a specific activity of 11.5 mA·cm^−2^ and the j_f_/j_b_ of 1.72 in an alkaline environment with good durability. These consequences provide new tactics for the designing and synthesis of non-precious metal electrocatalysts toward EOR in DEFCs.

## Data Availability

The data presented in this study are available on request from the corresponding author.

## Supplementary Materials

The following supporting information can be downloaded at: https://www.mdpi.com/article/10.3390/molecules28031173/s1, Figure S1: (a) XRD patterns of ZIF-67-1/2, ZIF-67-1/3, and ZIF-67-1/4; (b–d) SEM images of ZIF-67-1/2, ZIF-67-1/3, and ZIF-67-1/4. Figure S2: (a) XRD patterns of ZIF-67-1/4, NiCo-LDH-50, NiCo-LDH-100, and NiCo-LDH-150; (b–d) SEM images of NiCo-LDH-50, NiCo-LDH-100, and NiCo-LDH-150. Figure S3: (a) XRD patterns of ZIF-67-1/4, NiCo-LDH@CNTs-2%, NiCo-LDH@CNTs-2.5%, NiCo-LDH@CNTs-3%, and CNTs; (b–d) SEM images of NiCo-LDH@CNTs-2%, NiCo-LDH@CNTs-2.5%, and NiCo-LDH@CNTs-3%. Figure S4: EDS spectra of (a) NiCo-LDH-150, (b) NiCo-LDH@CNTs-2%, (c) NiCo-LDH@CNTs-2.5%, and (d) NiCo-LDH@CNTs-3%. Figure S5: XPS survey scan spectrum of NiCo-LDH-150, NiCo-LDH@CNTs-2%, NiCo-LDH@CNTs-2.5%, and NiCo-LDH@CNTs-3%. Figure S6: (a–f) The CV curves of NiCo-LDH@CNTs-3%, NiCo-LDH@CNTs-2.5%, NiCo-LDH@CNTs-2%, NiCo-LDH-150, ZIF-67-1/4, and Pd/C (20%) in 1.0 M KOH plus 1.0 M ethanol solution saturated with N_2_ at 20, 40, 60, 80, and 100 mV·s^−1^ sweep rate. Figure S7: CA curves of NiCo-LDH-150 and NiCo-LDH@CNTs-2.5% in 1.0 M KOH plus 1.0 M ethanol solution saturated with nitrogen. Figure S8: (a) XRD patterns, (b) Ni 2p XPS spectra, (c) Co 2p XPS spectra, (d) Ni K-edge XANES spectra, (e) Ni K-edge EXAFS oscillation functions k^3^χ(k), (f) the magnitude of k^3^-weighted FT of the Ni K-edge extended EXAFS spectra, (g) Co K-edge XANES spectra, (h) Co K-edge EXAFS oscillation functions k^3^χ(k), and (i) the k^3^-weighted FT magnitude of the Co K-edge extended EXAFS spectra of the NiCo-LDH@CNTs-2.5% and Re-NiCo-LDH@CNTs-2.5%. Figure S9: Proposed EOR reaction pathways on the NiCo-LDH surface of NiCo-LDH@CNTs-2.5%. Table S1: Comparison of physicochemical properties of NiCo-LDH@CNTs-3%, NiCo-LDH@CNTs-2.5%, NiCo-LDH@CNTs-2%, and NiCo-LDH-150. Table S2: Chemical compositions of the obtained NiCo-LDH@CNTs-3%, NiCo-LDH@CNTs-2.5%, NiCo-LDH@CNTs-2%, NiCo-LDH-150, and Re-NiCo-LDH@CNTs-2.5% extracted from XPS results. Table S3: Fitting impedance values of NiCo-LDH@CNTs-3%, NiCo-LDH@CNTs-2.5%, NiCo-LDH@CNTs-2%, NiCo-LDH-150, ZIF-67-1/4, and Pd/C (20%).

## Sample Availability

Samples of the compounds are available from the authors.
