# Supplementary material for: ZIF-67-Derived NiCo-Layered Double Hydroxide@Carbon Nanotube Architectures with Hollow Nanocage Structures as Enhanced Electrocatalysts for Ethanol Oxidation Reaction
Source: Molecules. 2023 Jan 25;28(3):1173. doi: 10.3390/molecules28031173 (PMC9920546; doi:10.3390/molecules28031173)
Supplement: Supplementary file 1 [file molecules-28-01173-s001.zip › molecules-2162193-supplementary.pdf]

Supporting Information

ZIF-67-derived NiCo-layered double hydroxide@carbon nanotubes architecture with hollow nanocages structure as enhanced electrocatalysts for ethanol oxidation reaction

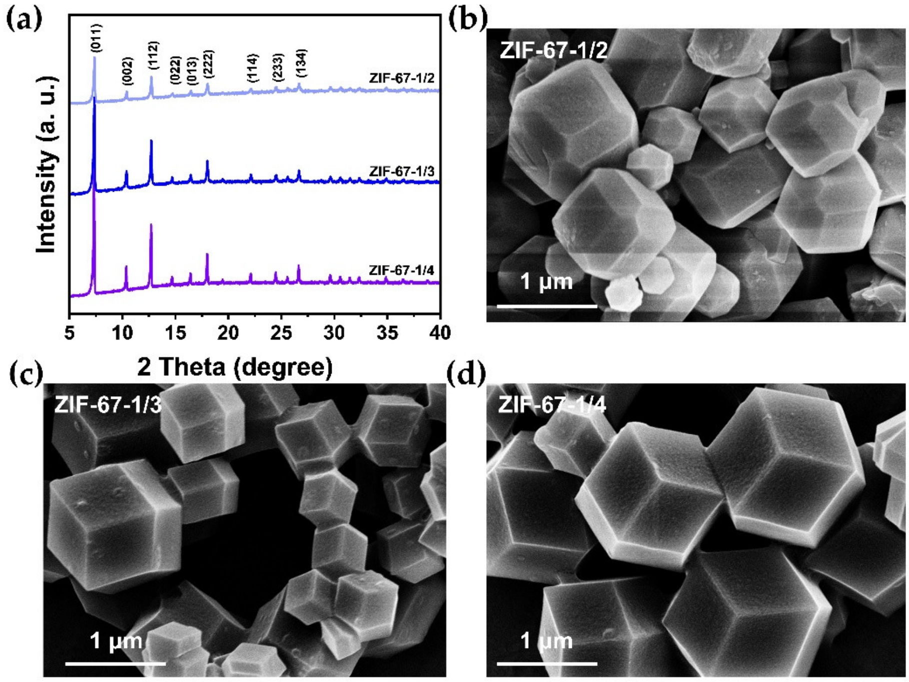

Figure S1. (a) XRD patterns of ZIF-67-1/2, ZIF-67-1/3, and ZIF-67-1/4, respectively; (b-d) SEM images of ZIF-67-1/2, ZIF-67-1/3, and ZIF-67-1/4, respectively.

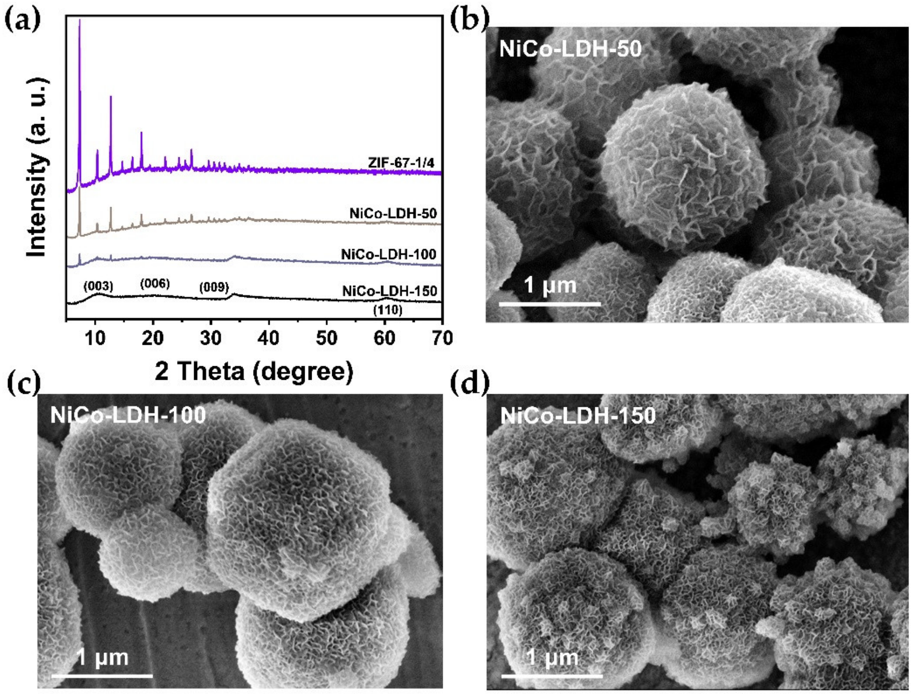

Figure S2. (a) XRD patterns of ZIF-67-1/4, NiCo-LDH-50, NiCo-LDH-100, and NiCo-LDH-150, respectively; (b-d) SEM images of NiCo-LDH-50, NiCo-LDH-100, and NiCo-LDH-150, respectively.

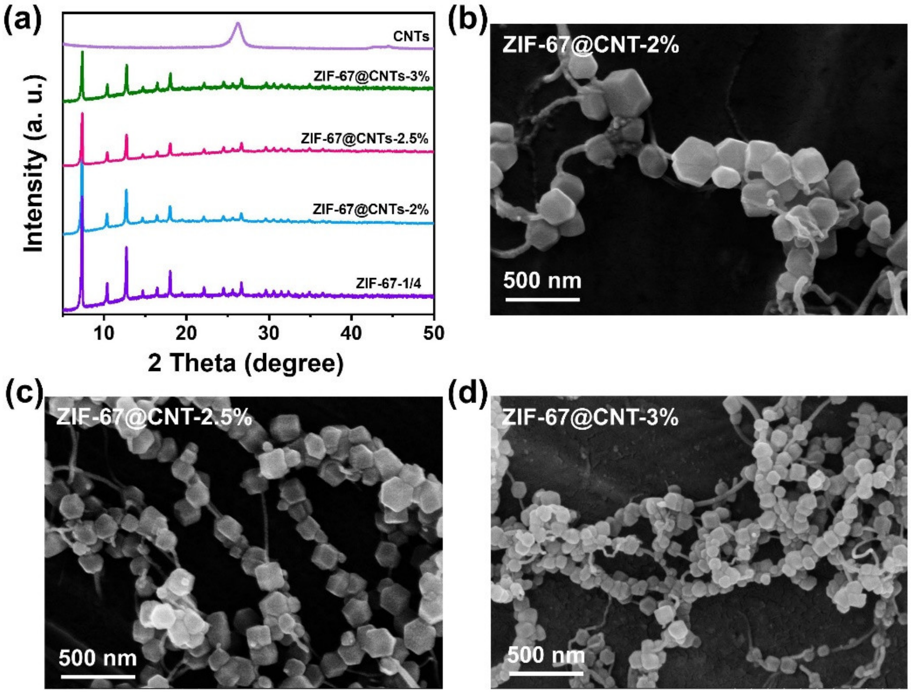

Figure S3. (a) XRD patterns of ZIF-67-1/4, ZIF-67@CNTs-2%, ZIF-67@CNTs-2.5%, ZIF-67@CNTs-3%, and CNTs, respectively; (b-d) SEM images of ZIF-67@CNTs-2%, ZIF-67@CNTs-2.5%, and ZIF-67@CNTs-3%, respectively.

Table S1. Comparison of physicochemical properties of NiCo-LDH@CNTs-3%, NiCo-LDH@CNTs-2.5%, NiCo-LDH@CNTs-2%, and NiCo-LDH-150

Samples	Surface area (m^2g^{-1})	Pore size (nm)	Pore volume (cm^3g^{-1})
NiCo-LDH@CNTs-3%	191.21	5.49	0.32
NiCo-LDH@CNTs-2.5%	140.89	6.09	0.26
NiCo-LDH@CNTs-2%	182.35	5.58	0.34
NiCo-LDH-150	22.12	6.92	0.04

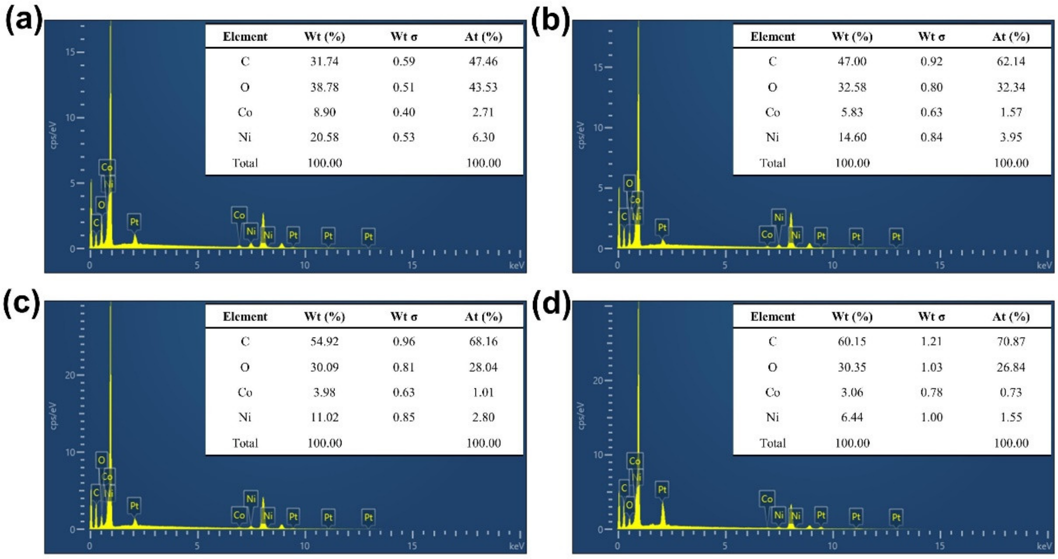

Figure S4. EDS spectra of (a) NiCo-LDH-150, (b) NiCo-LDH@CNTs-2%, (c) NiCo-LDH@CNTs-2.5%, and (d) NiCo-LDH@CNTs-3%, respectively.

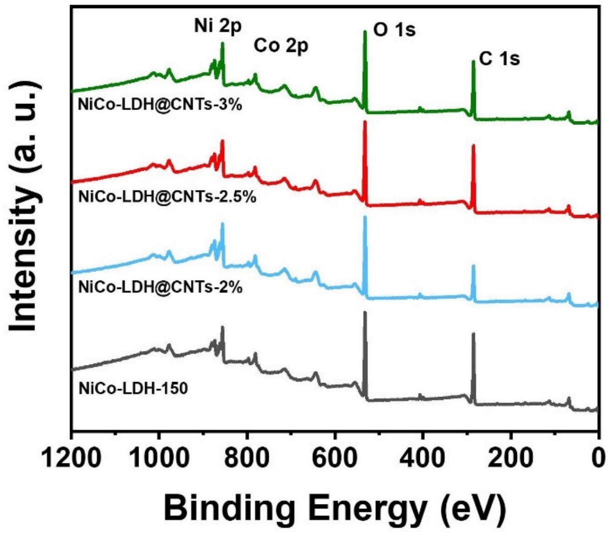

Figure S5. XPS survey scan spectrum of NiCo-LDH-150, NiCo-LDH@CNTs-2%, NiCo-LDH@CNTs-2.5%, and NiCo-LDH@CNTs-3%, respectively.

Table S2. Chemical compositions of the obtained NiCo-LDH@CNTs-3%, NiCo-LDH@CNTs-2.5%, NiCo-LDH@CNTs-2%, NiCo-LDH-150, and Re-NiCo-LDH@CNTs-2.5% extracted from XPS results

Samples	Ni^{3+}/Ni^{2+} ratio	Co^{3+}/Co^{2+} ratio
NiCo-LDH@CNTs-3%	0.43	1.45
NiCo-LDH@CNTs-2.5%	0.82	1.17
NiCo-LDH@CNTs-2%	0.65	1.38
NiCo-LDH-150	0.39	2.07
Re-NiCo-LDH@CNTs-2.5%	0.68	2.39

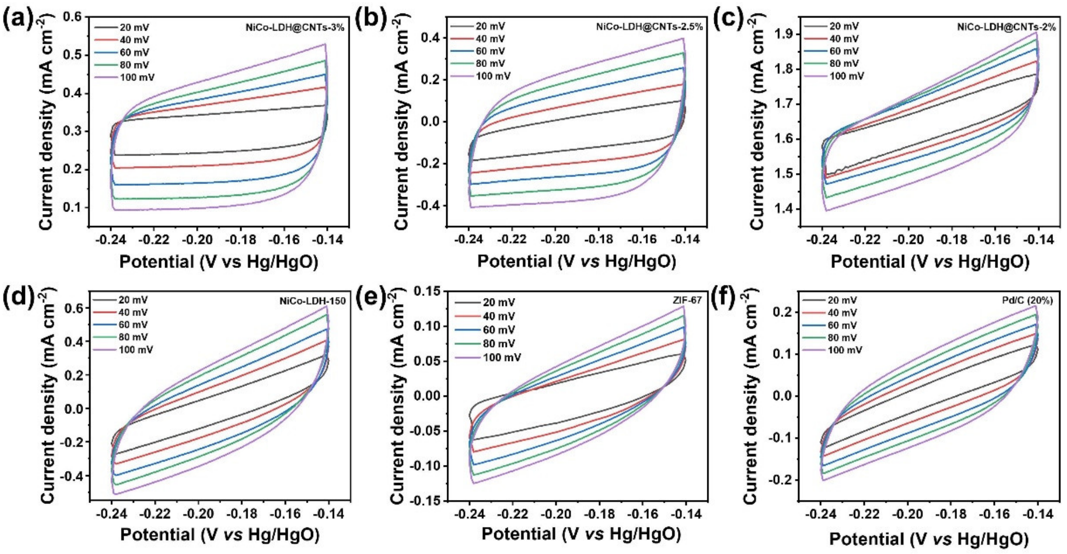

Figure S6. (a-f) The CV curves of NiCo-LDH@CNTs-3%, NiCo-LDH@CNTs-2.5%, NiCo-LDH@CNTs-2%, NiCo-LDH-150, ZIF-67-1/4, and Pd/C (20%) in 1.0 M KOH + 1.0 M ethanol solution saturated with N_2 at 20, 40, 60, 80, and 100 $mV{\cdot}s^{-1}$ sweep rate.

Table S3. Fitting impedance values of NiCo-LDH@CNTs-3%, NiCo-LDH@CNTs-2.5%, NiCo-LDH@CNTs-2%, NiCo-LDH-150, ZIF-67-1/4, and Pd/C (20%)

Samples	Rs (Ω)	Rct (Ω)
NiCo-LDH@CNTs-3%	3.03	37.26
NiCo-LDH@CNTs-2.5%	2.92	28.51
NiCo-LDH@CNTs-2%	3.10	35.11
NiCo-LDH-150	2.39	51.87
ZIF-67-1/4	2.66	99.86
Pd/C (20%)	1.91	96.86

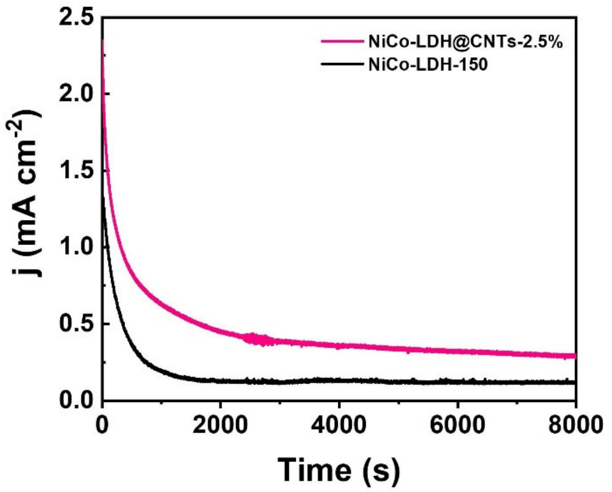

Figure S7. CA curves of the NiCo-LDH-150 and NiCo-LDH@CNTs-2.5% in 1.0 M KOH + 1.0 M ethanol solution saturated with nitrogen.

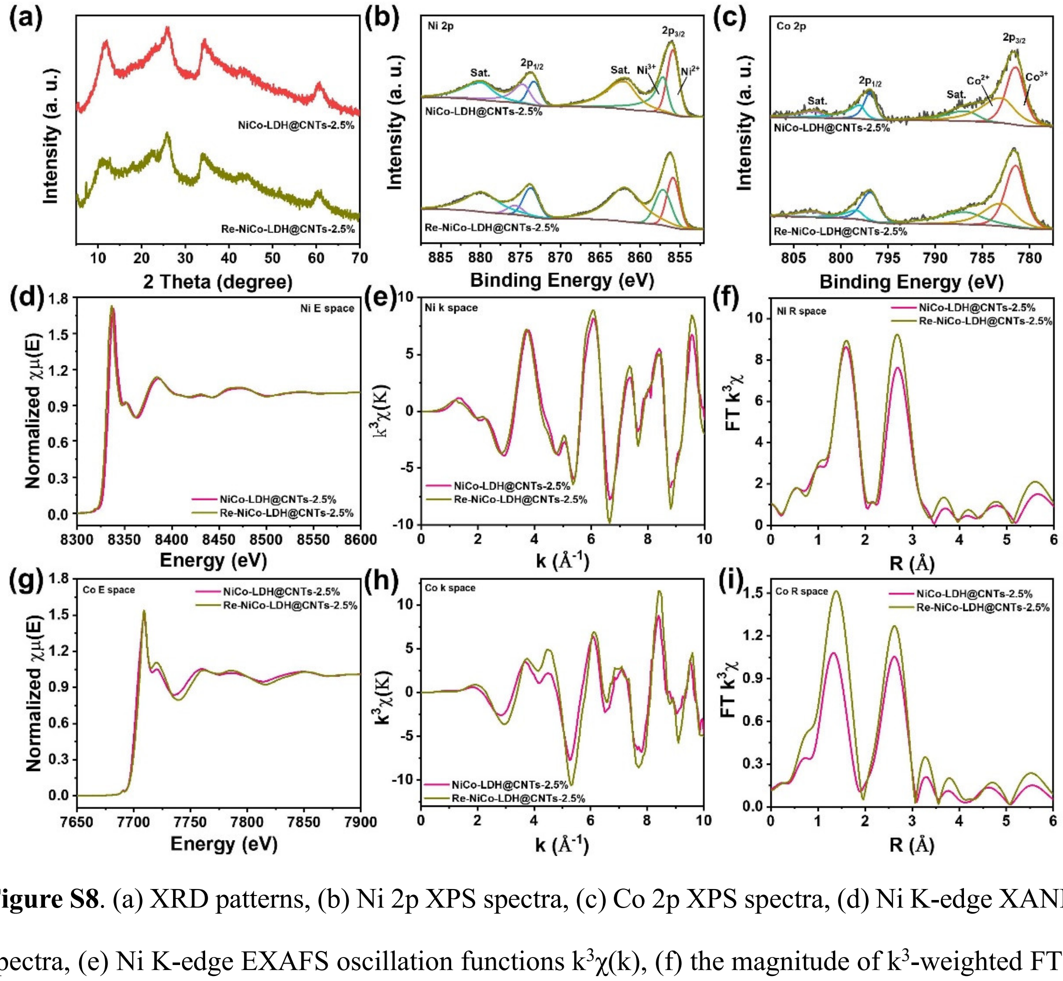

Figure S8. (a) XRD patterns, (b) Ni 2p XPS spectra, (c) Co 2p XPS spectra, (d) Ni K-edge XANES spectra, (e) Ni K-edge EXAFS oscillation functions $k^3\chi(k)$, (f) the magnitude of k^3-weighted FT of the Ni K-edge extended EXAFS spectra, (g) Co K-edge XANES spectra, (h) Co K-edge EXAFS oscillation functions $k^3\chi(k)$, and (i) the magnitude of k^3-weighted FT of the Co K-edge extended EXAFS spectra of the NiCo-LDH@CNTs-2.5% and Re-NiCo-LDH@CNTs-2.5%.

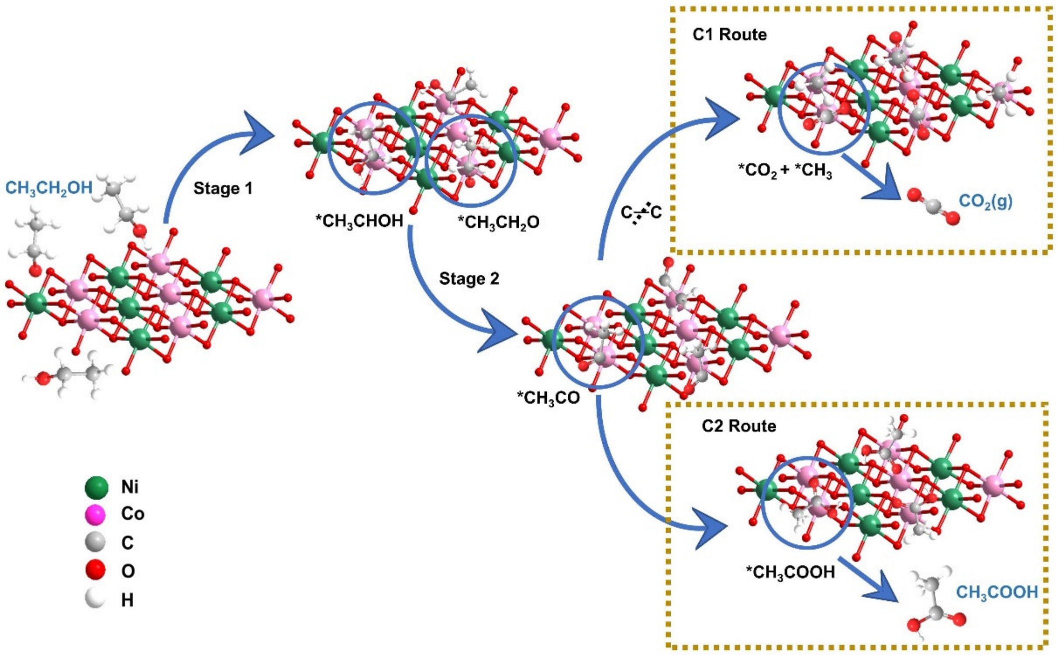

Figure S9. Proposed EOR reaction pathways on the NiCo-LDH surface of NiCo-LDH@CNTs-2.5%. The green, pink, silver, red and white balls represent nickel (Ni), cobalt (Co), carbon (C), oxygen (O), and hydrogen (H) atoms, respectively.
